# Glycated Haemoglobin Levels and Its Effect on Outcomes in Cardiac Surgery

**DOI:** 10.21470/1678-9741-2020-0188

**Published:** 2022

**Authors:** Daniyal Matin Ansari, Tinotenda Harahwa, Eyad Abuelgasim, Amer Harky

**Affiliations:** 1 St. George’s Hospital Medical School, London, United Kingdom; 2 Faculty of Medicine, Imperial College London, London, United Kingdom; 3 Department of Cardiothoracic Surgery, Liverpool Heart and Chest Hospital, Liverpool, United Kingdom; 4 Liverpool Centre for Cardiovascular Science, University of Liverpool and Liverpool Heart and Chest Hospital, Liverpool, United Kingdom

**Keywords:** Glycated Hemoglobin A, Comorbidity, Diabetes Mellitus, Morbidity, Cardiac Surgical Procedures, Renal Insufficiency, Wound Infection, Cardiovascular Diseases

## Abstract

There remains a significant paucity of information evaluating the effect of glycated HbA1c levels and its theorized effect on mortality and morbidity rates following cardiac surgery. Diabetes is a very common comorbidity in patients undergoing open heart surgery, as there is a shift in patient characteristics and greater risk. Currently, there is no clear consensus that an increase in HbA1c level is associated with increased perioperative mortality rate. However, the reported literature is more commonly able to demonstrate that elevated HbA1c levels is associated with increased rates of wound infection, cardiovascular events and renal failure, and thus, higher post-operative morbidities. This review aims to examine and synthesis the evidence behind each of the morbidities and mortalities associated with open heart surgery and the impact of high HbA1c on the reported outcomes.

**Table:** 

Abbreviations, acronyms & symbols			
ADA	= American Diabetes Association	LOS	= Length of stay
AF	= Atrial fibrillation	MI	= Myocardial infarction
AGEs	= Advanced glycation end-products	OCTOPUS	= Optimising Cardiac Surgery outcomes in People with diabetes
CABG	= Coronary artery bypass grafting	OPCAB	= Off-pump coronary artery bypass
CI	= Confidence interval	PCI	= Percutaneous coronary intervention
CMM	= Composite morbidity-mortality	TIA	= Transient ischemic attack
CVA	= Cerebrovascular accident	WHO	= World Health Organization
DM	= Diabetes mellitus	WMD	= Weighted mean difference
HbA1c	= Glycated haemoglobin		
HR	= Hazard ratio		

## INTRODUCTION

HbA1c levels refer to the levels of glycated haemoglobin present in the blood. These levels are used to represent the average plasma glucose of a patient over the previous 8 to 12 weeks^[1]^. HbA1c levels are commonly used to diagnose diabetes and are an indicator for the likelihood of patients with diabetes to develop diabetic complications. More recently, there has been a substantial amount of interest in using HbA1c levels as a screening test for those at high risk of developing diabetes^[2]^.

It is believed that HbA1c levels are viewed as a risk factor for post-operative complications of cardiac surgery. Cardiac surgery can result in increased glycated haemoglobin levels, which are associated with increased morbidity and mortality rates. Despite this, some studies suggest that increased HbA1c levels cannot be directly attributed as a cause of increased mortality and morbidity^[3]^. A recent meta-analysis of 7895 diabetic patients undergoing CABG surgery reported that higher HbA1c concentrations are frequently linked with the presence of other metabolic disorders, such as hypertension, dyslipidaemia and obesity, which are in fact the real problems causing an increased risk of poor clinical outcomes^[3]^.

However, a recent prospective, observational study of 7,565 inpatients showed that diabetes and elevated HbA1c levels were independently associated with a higher risk of adverse outcomes after surgery^[4]^.

The whole process behind surgical procedures can place a great deal of physical stress on patients and thus impair glucose metabolism, therefore worsening the diabetes status. Those suffering from diabetes and chronic hyperglycaemia, determined by their HbA1c levels, may be at particular risk of suffering from perioperative morbidity from diabetes-related complications, such as wound infections and renal dysfunction^[5,6]^.

This review aims to examine and synthesis the evidence behind each of the morbidities and mortalities associated with open heart surgery and the impact of high HbA1c on the reported outcomes.

### Literature Search

A comprehensive literature search was done on PubMed, SCOPUS, Embase, Cochrane, Google Scholar and Ovid databases to identify the articles that discussed HbA1c and its implications on perioperative cardiac surgery outcomes. The keywords used were ‘HbA1c’, ‘Glycated haemoglobin’, ‘Cardiac surgery’, ‘Haemoglobin A’, ‘HbA1c and outcomes’, ‘Diabetes’ and ‘Diabetic correlation’. The search terms were used as keywords and in combination as MeSH terms to maximize the output from literature findings. A staged literature search was done, whereby a separate literature search was performed for each section of this article and all the relevant studies were identified and summarized separately. If a study is reporting on many aspects of the practice of HbA1C, then the results have been shared between different parts of this review. The relevant articles are cited and referenced within each section separately. No limits were placed on publication time or language of the article.

### Pathophysiology of HbA1c

Proteins are frequently glycated during various enzymatic reactions when the conditions are physiologically favourable. However, in the case of haemoglobin A, the glycation occurs by a nonenzymatic reaction between the condensation of glucose and the N-terminal end of the β-chain of haemoglobin A, commonly lysine, forming a Schiff base (aldimine)^[6]^. The Schiff base may then undergo a rearrangement, converting into a stable Amadori product, otherwise known as HbA1c^[7]^. The formation of glycated haemoglobin is a routine part of the physiological function cycle. It was found that, as the average plasma glucose increases, so does the rate of this glycation reaction and the total quantity of HbA1c produced in the plasma^[8]^. The longer hyperglycaemia occurs in blood, the more glucose binds to haemoglobin in red blood cells. This results in increased rates of production of glycated haemoglobin. Once a haemoglobin molecule is glycated, it remains that way and its Amadori arrangement is viewed as “nearly irreversible”, according to a study by Higgins and Bunn^[9]^. As a result, this specific characteristic of haemoglobin is utilized as a biomarker, estimating average blood glucose levels in humans over the previous 2 to 3 months^[8]^.

The glycation of haemoglobin results in the formation of advanced glycation end-products (AGEs). Alongside their formation is the accompanied release of free radicals and oxidants as side products from the Amadori rearrangement. This result in oxidative damage to cells and the extracellular matrix of the body tissue^[10]^. It is found that, additionally, the accumulation of these free radicals alters the erythrocyte membrane properties, leading to erythrocyte aggregation, increased blood viscosity and impaired blood flow^[11]^. This can result in shear stress, due to the thicker abrasive blood consistency, which affects the vascular endothelium and can result in a number of inflammatory and atherogenic events if the levels become excessive^[12]^. Following a consultation from the World Health Organization (WHO), it was concluded that HbA1c can be used as a viable diagnostic test for diabetes. This was as long as “stringent quality assurance tests are in place” and assays are kept consistent and standardised to their stated criteria, aligned to their internationally set reference values^[13]^. It is also important that there are no conditions present that interfere with the accuracy of its measurement. An HbA1c of 6.5% is recommended as the cut-off point for diagnosing diabetes, according to the guidelines set by the World Health Organization^[13]^. A value <6.5% does not exclude diabetes diagnosed using glucose tests^[13]^. According to the American Diabetes Association (ADA) 2020 Guidelines, the HbA1c value should be kept below 7% in all non-pregnant adult diabetics (53 mmol/mol)^[14]^. Values >7% indicate an increased chance of progression to diabetic complications, especially microvascular ones. The HbA1c levels appear to be closely related to blood glucose levels and are resultantly affected by any forms of glycaemic control. An initially raised HbA1c level has been found to progressively decrease in the weeks following the introduction of insulin and dietary therapy, with a tendency for these values to level out after approximately seven weeks of therapy^[15]^.

### HbA1c and Mortality Rates

As the incidence of diabetes mellitus (DM) increases, the proportion of people with DM undergoing cardiac surgery has also increased^[16]^. There is a large body of evidence that has looked at the association between patient HbA1c levels and mortality following cardiac surgery. The evidence on the effect of HbA1c levels on mortality is contradictory, with some studies reporting that increased HbA1c levels are associated with increased mortality^[17,18]^. But by in large, most studies seem to show that increased HbA1c is not predictive of increased mortality as a solo indicator^[19-24]^. The findings from all these studies are summarised in Table 1 and Table 2.

**Table 1:** Summary of clinical outcomes in included studies that showed that the HbA1c level is predictive of mortality.

Study	Study design	Single/ Multi-centre	Sample size	Main outcomes
Kuhl et al.^[17]^, 2016	Retrospective	Multi-centre (across Sweden)	6,313 patients	•HbA1c associated with increased risk of death in patients with HbA1c levels 9.1-10.0% (HR 1.26, 95% CI 1.04-1.53)
				•HbA1c associated with increased risk of death in patients with HbA1c >10.0% (HR 1.33, 95% CI 1.05-1.69)
				•Stepwise increased risk of death with increasing HbA1c: 8.1-9.0% (HR 1.17, 95% CI 1.04-1.33), 9.1-10.0% (HR 1.44, 95% CI 1.22-1.70) and >10.0% (HR 1.50, 95% CI 1.22-1.84)
Halkos et al.^[18]^, 2008	Retrospective	Single centre (USA)	3,089 patients	•Elevated HbA1c predicted in-hospital mortality after CABG (OR 1.40 per unit HbA1c increase, 95% CI 1.06-1.86, *P*=0.019)
				•HbA1c >8.6% associated with a 4-fold increase in mortality
Kim et al.^[19]^, 2020	Retrospective	Single centre (South Korea)	703 patients	•Incidence of the composite post-operative morbidity and mortality endpoint was greater in patients with HbA1c ≥7.0% when compared to HbA1c <7.0% (21% *vs*. 15%, *P*=0.041)
				•·A greater pre-operative HbA1c ≥7.0% was independently associated with composite mortality and morbidity endpoints (OR 1.60, 95% CI 1.03-2.50, *P*=0.038)

CABG=coronary artery bypass grafting; CI=confidence interval; HR=hazard ratio; OR=odds ratio

**Table 2:** Summary of clinical outcomes in included studies that showed that the HbA1c level had no effect on post-operative mortality.

Study	Study design	Single/ Multi-centre	Sample size	Main outcomes
Narayan et al.^[20]^, 2017	Retrospective	Single centre (India)	4,678 patients	•30-day mortality was significantly higher in patients with HbA1c ≥6.5% when compared to those with HbA1c <6.5% (4.22% *vs*. 3.07%, *P*=0.0035), but in the multivariable analysis there was no significant difference in mortality (OR 1.36, 95%, CI 0.95-1.953, *P*=0.08)
van den Boom et al.^[21]^, 2018	Retrospective	Single centre (USA)	6,393 patients	•HbA1c was found to not be a significant predictor of post-operative mortality in cardiac surgery (*P*=0.88)
Tsuruta et al.^[22]^, 2011	Prospective	Single centre (Japan)	306 patients	•No significant difference in all-cause mortality or cardiac mortality between patients with HbA1c <6.5%, 6.5-7.5% and ≥7.5% (HR 0.80, 95% CI 0.49-1.23, *P*=0.26 and *P*=0.17, respectively)
				•Multivariate analysis showed that HbA1c did not predict mortality
Ramadan et al.^[23]^, 2018	Prospective	Single centre (Egypt)	80 patients	•There was no statistically significant difference for in-hospital mortality between patients with HbA1c ≤7.0% and HbA1c >7.0% (2.5% *vs*. 7.5%, *P*=0.608)
				•There was no statistically significant difference for one-year mortality between patients with HbA1c ≤7.0% and HbA1c >7.0% (5.13% *vs*. 8.11%, *P*=0.600)
Subramaniam et al.^[24]^, 2014	Prospective	Single centre (Israel)	1,461 patients	•No statistically significant difference in the incidence of death between patients with HbA1c <6.5% and with HbA1c ≥6.5% (3% *vs*. 2.6%, *P*=0.90)
Biskupski et al.^[28]^, 2014	Retrospective	Single centre (Poland)	350 patients	•There was no significant difference in the incidence of death between patients with HbA1c <7.0% compared to HbA1c 7.0-8.0% (1.02% *vs*. 2.27%, *P*=0.78)
				•There was no significant difference in the incidence of death between patients with HbA1c <7.0% and with HbA1c >8.0% (1.02% *vs*. 4.74%, *P*=0.20)
Finger et al.^[30]^, 2017	Retrospective	Single centre (USA)	531 patients+D21:D24D19:D24	•There was no statistically significant difference in 30-day mortality between patients with HbA1c ≤7.0% or with HbA1c >7.0% (2.74% *vs*. 3.51%, *P*=0.670)

CI=confidence interval; HR=hazard ratio; OR=odds ratio

The study with the largest patient cohort looked at outcomes in 6,313 patients with type 2 DM who underwent CABG surgery between 2003 and 2013^[17]^. The study found that HbA1c was associated with an increased risk of death in patients with an HbA1c level between 9.1 and 10.0% (hazard ratio [HR] 1.26, 95% CI 1.04-1.53), and this risk was even greater in patients with HbA1c >10.0% (HR 1.33, 95% CI 1.05-1.69). One study showed that 30-day mortality was significantly higher in patients with HbA1c >6.5% compared to those with Hb1ac <6.5% (4.22% *vs*. 3.07%, *P*=0.0035), however, following multivariable adjustment, this association was lost and there was no significant difference in the mortality rates^[20]^.

All of these studies, overall, demonstrate that increased HbA1c increases mortality in cardiac surgery patients and all of them, with the exception of one, had large sample sizes, meaning they were all by in large sufficiently statistically powered to make their findings significant. However, all the aforementioned studies were retrospective in nature, meaning they were liable to biases inherent in the study design and the studies do not demonstrate causality.

The largest cohort study demonstrating that there is no relationship between HbA1c levels and mortality looked at the outcomes in 6,393 patients undergoing cardiac surgery^[21]^. The study found that HbA1c was not a significant predictor of post-operative mortality (*P*=0.88). Further studies similarly showed that there was no significant difference in mortality following cardiac surgery according to HbA1c levels, regardless of the HbA1c cut-off levels utilised^[22,23]^.

Overall, the evidence showing that there is no significant difference between higher HbA1c levels in the incidence of mortality outweighs the evidence showing that elevated HbA1c is associated with increased mortality, suggesting that HbA1c alone may not be predictive of mortality following cardiac surgery.

### HbA1c and Wound Infection

A chronic state of impaired glucose metabolism has long been demonstrated to affect components of the immune system, thereby meaning that HbA1c may impact the rate of post-operative infections. Post-operative wound infection affects patient morbidity and hospital length of stay following cardiac surgery and hence it is important to analyse the relationship between HbA1c levels and the infection rate. Some studies have shown that HbA1c level has no effect on the rate of wound infection^[22-26]^. By contrast, there are studies that have demonstrated that elevated HbA1c levels are associated with an increased rate of wound infection^[18,20,27]^. The main outcomes of these studies are summarised in Table 3 and Table 4.

**Table 3:** Summary of clinical outcomes in included studies that showed that the HbA1c level had no effect on post-operative wound infection.

Study	Study design	Single/ Multi-centre	Sample size	Main outcomes
Biskupski et al.^[28]^, 2014	Retrospective	Single centre (Poland)	350 patients	•There was no significant difference in the incidence of wound infection between patients with HbA1c <7.0% compared to HbA1c 7.0-8.0% (1.53% *vs*. 3.40%, *P*=0.57)
				•There was no significant difference in the incidence of wound infection between patients with HbA1c <7.0% and with HbA1c >8.0% (1.53% *vs*. 5.97%, *P*=0.13)
Tsuruta et al.^[22]^, 2011	Prospective	Single centre (Japan)	306 patients	•There was no significant difference in the frequency of mediastinitis between patients with HbA1c <6.5%, HbA1c 6.5-7.5% and HbA1c ≥7.5% (*P*=0.11)
Ramadan et al.^[23]^, 2018	Prospective	Single centre (Egypt)	80 patients	•There was no statistically significant difference in the incidence of deep sternal wound infection between patients with HbA1c ≤7.0% and >7.0% (0 *vs*. 5%, *P*=0.152)
Göksedef et al.^[29]^, 2018	Prospective	Single centre (Turkey)	150 patients	•HbA1c level was found to not affect the short-term infectious complication rates

There were no statistical differences between patients with HbA1c <7.0% and HbA1c >7.0% for non-sternal infection (2.1% *vs*. 3.5%, *P*=0.744) and for deep sternal wound infection (*P*=0.431)

**Table 4:** Summary of clinical outcomes in included studies that showed that the HbA1c level is predictive of post-operative wound infection.

Study	Study design	Single/ Multi-centre	Sample size	Main outcomes
Narayan et al.^[20]^, 2017	Retrospective	Single centre (India)	4,678 patients	•HbA1c ≥6.5% was found to be associated with a higher rate of sternal dehiscence (0.44% *vs*. 1.27%, *P*=0.002) and deep sternal wound infection (0.53% *vs*. 1.32%, *P*=0.004) compared to HbA1c <6.5% •Multivariable analysis found that HbA1c ≥6.5% is an independent risk factor for sternal dehiscence (OR 2.161, 95% CI 1.008-4.630, *P*=0.04)
Subramaniam et al. ^[24]^, 2014	Prospective	Single centre (Israel)	1,461 patients	•Rate of deep sternal wound infection was significantly higher in patients with HbA1c ≥6.5% than <6.5% (2.2% *vs*. 0.5%, *P*=0.008)
Finger et al.^[30]^, 2017	Retrospective	Single centre (USA)	531 patients	•HbA1c >7.0% was associated with increased risk of infection compared to patients with HbA1c ≤7.0% (10.76% *vs*. 31.58%, *P*<0.001) •HbA1c >7.0% was associated with increased risk of sternal wound infection (0.84% *vs*. 5.26%, *P*=0.030)
Gatti et al.^[25]^, 2016	Prospective	Multi-centre (16 centres across France, Italy, Germany, UK, Finland and Sweden)	114 patients	•Baseline HbA1c was found to be significantly higher in patients who developed sternal wound infection than those who did not (mean 54±17 *vs*. 45±13 mmol/mol, *P*<0.0001) •Logistic regression analysis showed that HbA1c was an independent risk factor for sternal wound infection (OR 1.04, 95% CI 1.02-1.05, *P*<0.0001) •HbA1c >8.6% was associated with the highest risk of sternal wound infection (adjusted OR 5.01, 95% CI 2.47-10.15) •HbA1c >7.0% was found to be associated with increased incidence for superficial wound infection (7.2% *vs*. 1.6%), deep wound infection (2.4% *vs*. 1.7%) and mediastinitis (1.1% *vs*. 0.6%) when compared to HbA1c ≤7.0% (*P*<0.0001) •HbA1c >7.0% was associated with an increased risk of sternal wound infection (OR 2.15, 95% CI 1.28-1.96, *P*=0.004)

CI=confidence interval; HR=hazard ratio; OR=odds ratio

Biskupski et al.^[28]^ analysed outcomes in 350 patients who were stratified into three groups according to HbA1c levels: <7.0%, 7.0-8.0%, and >8.0%. The study found that there was no significant difference in the incidence of wound infection in the group with HbA1c <7.0% when compared to those with HbA1c 7.0-8.0% and to those with HbA1c >8.0% (1.53% *vs*. 3.40%, *P*=0.57 and 1.53% *vs*. 5.97%, *P*=0.13, respectively). In a study in which the primary outcome analysed was the incidence of wound infections, it was found that there was no statistically significant difference in the rate of all subset of wound infection in patients with HbA1c <7.0% and in those with HbA1c >7.0% (*P*=0.431 and *P*=0.744 for sternal and non-sternal infections, respectively)^[29]^.

By contrast, Gatti et al.^[25]^ specifically looked at whether HbA1c was a risk factor for sternal wound infection following CABG surgery in 2,130 patients and found that the mean baseline HbA1c level was significantly higher in patients who had sternal wound infection (54±17 mmol/mol *vs*. 45±13 mmol/mol; *P*<0.0001). Logistic regression showed that the HbA1c level was an independent risk factor for sternal wound infection (OR 1.04, 95% CI 1.02-1.05, *P*<0.0001) with an HbA1c level >8.6% associated with the highest risk of sternal wound infection (OR 5.01, 95% CI 2.47-10.15). Similar results were shown in a range of other studies that all showed that pre-operative elevated HbA1c was associated with an increased incidence of both sternal and non-sternal wound infection.

Overall, the evidence suggests that elevated HbA1c level is associated with an increased incidence of wound infection in cardiac surgery patients, but it is unclear if there is a specific level of HbA1c associated with this increased incidence that could serve as a target for pre-operative glycaemic control as studies used a range of HbA1c cut-offs in their analyses. Yet, further work is required to determine the exact cut-off level and the association between HbA1c level and non-sternal wound infections.

### Cardiovascular Events with Poorly Controlled HbA1c

Several studies have analysed the relationship between HbA1c levels and the incidence of cardiovascular events, such as peri-operative myocardial infarction (MI), atrial fibrillation (AF), and low cardiac output syndrome. The majority of literature, including a large retrospective study with nearly 4,000 participants, has demonstrated that the HbA1c level alone cannot be used as a predictor for cardiovascular events^[23]^. By contrast, only one study showed a relationship between HbA1c and cardiovascular event, which suggested that elevated pre-operative HbA1c may be protective for the development of AF^[30]^.

The largest cohort study that demonstrated that there was no relationship between the HbA1c level and cardiovascular events analysed outcomes in 1,461 patients undergoing CABG with or without valvular surgery^[25]^. The study found that there was no difference in the incidence of cardiovascular events in patients with HbA1c <6.5% and >6.5%: AF (26.3% *vs*. 26.6%, *P*=0.90), acute MI (0.5% *vs*. 0, *P*=0.333) and cardiac tamponade (0 *vs*. 0.2%, *P*=0.313). Other studies similarly showed that there was no significant difference in the incidence of cardiovascular events between patients with high HbA1c levels and those with normal levels^[27,30,32]^.

Kinoshita et al.^[30]^ carried out a retrospective analysis of 912 patients who underwent isolated CABG. They found that the median HbA1c was significantly lower in patients who developed AF post-operatively when compared to patients who did not (5.8%, 95% CI 5.4-6.3 *vs*. 6.1%, 95% CI 5.5-7.2, *P*=0.01). Additionally, results also showed that the incidence of post-operative AF showed a stepwise trend in which the incidence decreased as the HbA1c level decreased: incidence of 28.3% for patients with HbA1c ≤5.6%, 17.4% for HbA1c 5.7-6.7% and 12.5% for HbA1c 6.8-11.4% (*P*=0.01). These findings suggest that high HbA1c levels may be associated with a lower risk of post-operative AF, but this is a single retrospective study carried out in Japanese patients, thereby limiting the generality of findings and cannot be used as a sole evidence to support this clinical outcome in the general population.

### HbA1c and Cerebrovascular Accident

Cerebrovascular accident (CVA) is a severe complication following CABG surgery. CVA indicates whether a patient had a stroke (acute neurological deficit lasting more than 24 hours) or a transient ischemic attack (TIA) (deficit resolving within 24 hours). The meta-analysis by Zheng et al. assessed the effect of HbA1c levels and CVA among diabetic patients (n=4,356) undergoing CABG surgery^[31]^. Their analysis, which included five studies^[18,22,31-33]^, indicated that HbA1c levels were directly correlated with the risk of stroke after CABG surgery (OR 2.07, 95% CI 1.29-3.32, *P*=0.003), with very low heterogeneity (I^^2^^=0%, *P*=0.42). Only one retrospective study significantly indicated a possible role for HbA1c in predicting stroke outcomes^[18]^. This retrospective study had a comparatively large sample size (n=3,089), hence sufficient power to solely elucidate an association between HbA1c levels and stroke. The study indicated that patients with HbA1c values of 7.6% or more have adjusted odds of CVA 2.23 (1.06-4.70) times higher than patients with values below this threshold. The overall incidence of stroke for all patients was very low (1.7%). Other studies were either contradictory^[31,32]^ or inconclusive^[22,33]^ due to small sample sizes and inherent low incidence of stroke.

However, a larger and more recent meta-analysis (n=5,381) conducted by Wang et al.^[34]^ showed that there was no significant difference in stroke incidence between diabetic patients with lower pre-operative HbA1c levels and those with higher pre-operative HbA1c levels after CABG and PCI (OR 1.49, 95% CI 0.94-2.37, *P*=0.37, and I^^2^^=8%). Higher HbA1c levels were defined as pre-operative HbA1c ≥6.5% or 7% and lower HbA1c levels as pre-operative HbA1c <6.5% or 7%.

Interestingly, Biskupski et al.^[28]^ noted that TIAs were more common in patients with HbA1c <7%, while strokes were significantly more common in patients with decompensated diabetes (HbA1c <7% *vs*. HbA1c >8%, *P*=0.04). Current research indicates a potential association between the baseline risk of TIA events and exposure to hypoglycaemia^[35,36]^.

### HbA1c and Renal Failure

A recent prospective study reported that acute renal failure is one of the most common post-CABG complications in diabetic patients^[36]^. Compared with diabetic patients with HbA1c ≤7%, those with HbA1c >7% had more incidence of renal failure (10% *vs*. 0%)^[33]^. Additionally, the results of a recent meta-analysis, involving nine studies (n=5,858), suggested that a higher pre-operative HbA1c level was associated with a high risk of renal failure after cardiac surgery (OR=1.63, 95% CI 1.13-2.33, *P*=0.47, and I^2^=0%)^[34]^. Furthermore, by using receiver operating characteristic value thresholds, Halkos et al.^[18]^ showed that renal failure occurred more commonly in patients with elevated HbA1c (threshold 6.7, OR 2.1). It is worth noting that the 6.7% threshold derived is below the 7% glycaemic target of the American Diabetes Association^[14]^.

### HbA1c and Prolonged Hospital Stay

There are differences between studies in the definition of a prolonged hospital stay (from ≥3 to 14 days)^[37,38]^, which sheds an interesting light on the association between HbA1c and hospital length of stay (LOS).

In a retrospective study (n=570), extended LOS was defined as >3 days^[11]^. The authors found that HbA1c was an independent predictor of hospital stay, regardless of blood sugar levels (*P*=0.001). Moreover, Medhi et al.^[37]^ found similar results of LOS in 135 patients who underwent coronary artery bypass surgery; HbA1c ≥7% was found to be a strong predictor of LOS ≥6 days (*P*=0.025). However, interestingly, when defining prolonged LOS as ≥14 days, LOS was not affected by pre-operative HbA1c levels (with a cut-off of HbA1c=7% for optimal and suboptimal levels, *P*=0.367)^[38]^.

It is noted that, in diabetic patients undergoing CABG, HbA1c levels significantly correlated with post-operative LOS. Patients with suboptimal medium-term glycaemic control (HbA1c >7%, n=38) had longer LOS than patients with optimal medium-term glycaemic control (HbA1c ≤7%, n=57) (mean post-operative LOS 6 days *vs*. 7.5 days; post-operative LOS mean rank: 32 days *vs*. 46 days; *P*=0.008)^[33]^.

The meta-analysis of five studies (n=3,002) conducted by Wang et al.^[34]^ reported a higher pre-operative HbA1c level resulted in a 1.08-day mean increase in hospital stay after cardiac surgery (WMD=1.08, 95% CI 0.46-1.71).

On the other hand, intensive care stay was not affected by the level of HbA1c, as several studies reported no significant difference in intensive care unit days between patients with lower pre-operative HbA1c levels and those with higher HbA1c levels after cardiac surgery^[27,32,34,40]^.

### Summary

Despite conflicting clinical evidence on higher HbA1c as a prognostic marker of poor outcomes after cardiac surgery, there is universal consensus of possible underlying mechanism of association. Therefore, future research is needed to further elucidate any possible clinical association is warranted. Such research has the potential to improve cardiac surgery clinical practice guidelines.

HbA1c has a significant role in inducing dyslipidaemia, hyperhomocysteinemia, hypertension and increased C-reactive protein, oxidative stress, and blood viscosity^[11]^. Cardiac surgery, stress, and anaesthesia can exacerbate oxidative stress and increase blood viscosity, thereby perpetuating the effect of high HbA1c in patients with diabetes and the likelihood of devolvement of cardiovascular event^[41]^. Increased blood viscosity of diabetic patients, leading to blood clots, can precipitate acute MI^[42]^. Moreover, high HbA1c can cause vascular endothelial cell damage due to shear stress from blood flow, with increased cellular proliferation^[12]^, which can cause MI and stroke after coronary artery stenting^[43]^. Chronic hyperglycaemia-induced dysmetabolism weakens chemokine chemotaxis and decreases immune function in patients with diabetes^[43]^. This increases the likelihood of wound infection after cardiac surgery and increases collateral tissue damage upon infection. For these reasons, high pre-operative HbA1c levels may be predictive of a prolonged post-operative hospital stay.

### Future Directions

In this review, we have discussed both retrospective and prospective studies that have looked at HbA1c as a predictor of various post-operative adverse outcomes. However, most of these studies had small sample sizes, thus, from the onset, limited ability to draw statistically meaningful conclusions about adverse outcomes of inherently low event rates in any cohort; reduced statistical power and possible type II statistical error. Moreover, studies have used different cut-off values of pre-operative HbA1c levels, such as 6.5%, 7%, 7.5%, and 8%. Further research should be directed at determining a pre-operative cut-off of ‘suboptimal glycaemic control’ for pre-operative optimisation clinical guidelines of the surgical patient.

A recent retrospective study by Kim et al.^[19]^ of 703 patients with diabetes mellitus who underwent off-pump coronary artery bypass surgery (OPCAB) provides the strongest evidence to date of the prognostic role of HbA1c. The use of composite post-operative morbidity and mortality (CMM) endpoints (permanent stroke, prolonged ventilation, deep sternal wound infection, renal failure, reoperation, and 30-day mortality) attenuates the prospect of a misleading statistical conclusion by combining adverse events of low incidence. Kim et al.^[19]^ found that the incidence of CMM endpoints was greater in patients with HbA1c ≥7.0% (21% *vs*. 15%, *P*=0.041). Moreover, receiver operator characteristic curve analysis revealed HbA1c 7.85% as the optimal threshold for CMM endpoints (area under the curve 0.556, 95% CI 0.501-0.611, *P*=0.048). This study has provided rationale for future prospective studies with sufficient power to examine whether postponing cardiac surgery in patients with high pre-operative HbA1c levels would improve post-operative outcomes.

Moreover, Kim et al.^[19]^ indicated that a high pre-operative HbA1c (≥7.0%) level alone, and not the variables related to perioperative glycaemic control, was independently associated with adverse outcome in diabetic patients undergoing OPCAB, although high HbA1c levels contributed to greater perioperative glycaemic variability. However, a randomised controlled trial conducted by Bláha et al.^[44]^ suggested that it is cardiac surgery patients with previously undiagnosed diabetes who have the worst prognosis. Comparable conclusion was suggested in non-cardiac studies^[45,46]^. Recent studies have shown that perioperative intravenous insulin infusion is more frequently administered in known diabetics due to more frequent monitoring of their capillary glucose concentrations^[19,47]^. However, despite more frequently administered insulin in the high HbA1c group, adverse outcomes remained more prevalent in this group compared to normal HbA1c group, thereby further attenuating the prognostic role of HbA1c. Nevertheless, optimisation of pre-operative HbA1c concentrations with a combined intravenous and subcutaneous insulin glucose has been shown to reduce surgical mortality and morbidity in diabetic patients undergoing cardiac surgery^[48]^.

Future research should be directed at the determining the optimal level of perioperative glycaemic management and the crucial perioperative period to maintain this HbA1c level. Although there are current ongoing outcome studies currently in this area (*e.g*. the Optimising Cardiac Surgery outcomes in People with diabetes (OCTOPUS) trial - protocol number HTA16/25/12), there remain few data on the outcomes and effects of intervention on those who are not known to have diabetes^[49-51]^.

## CONCLUSION

The cohorts of patients undergoing cardiac surgery are shifting to a higher risk than a decade ago, with more diabetes and other comorbidities; therefore, poorly controlled diabetes and deranged pre-operative HbA1c can have a detrimental effect on outcomes after cardiac surgery and significantly affect morbidity. Optimum peri-operative diabetes control can help to minimize post-operative complications.
